# Retrieval of a migrated biliary stent using a customized goose-neck snare

**DOI:** 10.1055/a-2686-3583

**Published:** 2025-09-11

**Authors:** Kazuya Sumi, Hisaki Kato, Yukiko Okada, Yuki Kawasaki, Jun Ushio, Takayoshi Ito, Haruhiro Inoue

**Affiliations:** 1 378609 Digestive Diseases Center, Showa Medical University Koto-Toyosu Hospital, Tokyo, Japan

Stents are commonly used to treat acute cholangitis (AC) and obstructive jaundice caused by strictures of the bile duct (BD) and pancreatic duct. Although rare, stent migration can occur. Stent retrieval is often attempted using forceps, baskets, or balloon catheters; however, the efficacy and reliability of these methods are limited, rendering the retrieval challenging. Several alternative techniques have been reported, including cases where metal and plastic stents (PS) were involved 1 2 3 4 .

A woman in her 80s presented with abdominal pain and was diagnosed with AC. Endoscopic retrograde cholangiopancreatography (ERCP) was planned. She had a history of cholangitis several years earlier, during which stone extraction was difficult due to multiple large common bile duct stones, and a PS was placed ( Fig. 1 ). In the initial ERCP, removal with grasping forceps failed due to firm adhesion to stones, resulting in stent fracture. Another PS was inserted for drainage, and a second ERCP was planned.

**Fig. 1:**
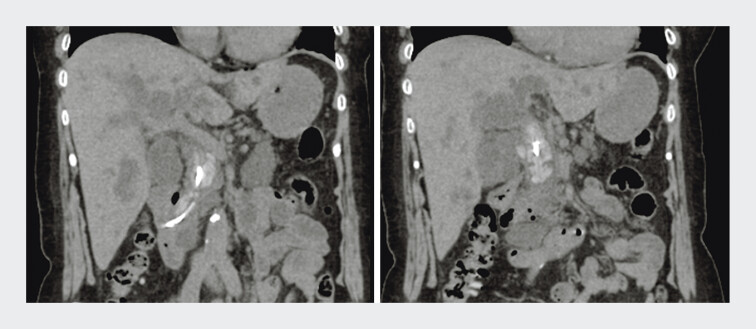
Multiple large common bile duct (BD) stones surrounding a plastic stent were observed.

After removing the second PS, the initial one was found to have completely migrated. Given its firm adhesion, strong traction was necessary for retrieval.

As the proximal BD was filled with multiple large stones, it was necessary for the stent to be grasped in the BD’s open space using a customized gooseneck-shaped snare. To maintain reliable bending and axial control, the tip of a rotatable snare was fixed to the sheath using nylon thread ( Fig. 2 ). The snare was inserted over a guidewire and deployed, allowing strong traction with easy grasping and successful PS removal ( Fig. 3 , Fig. 4 , Video 1 ).

**Fig. 2:**
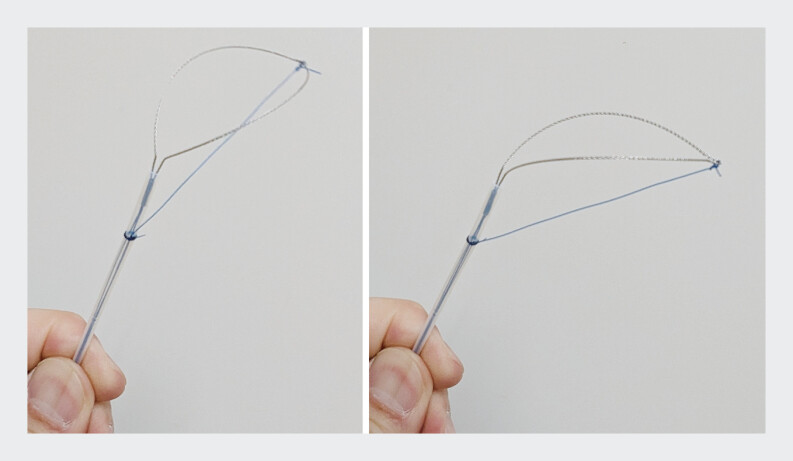
The snare’s tip was tied to the sheath with a nylon thread. Its rotatable design allows for easy adjustment of direction.

**Fig. 3:**
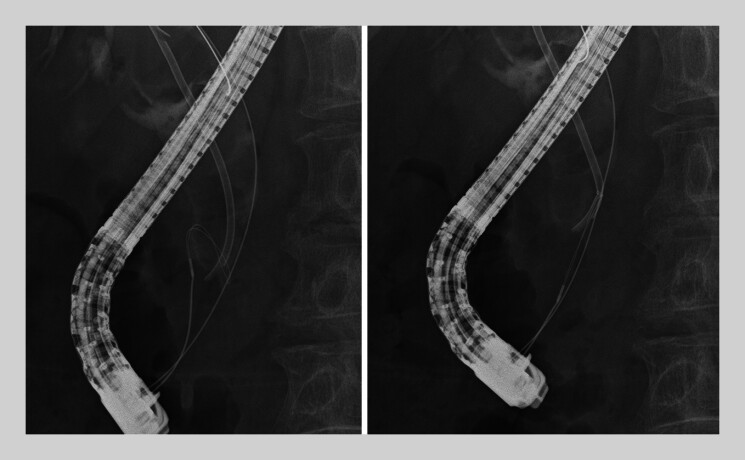
A customized goose-neck snare was able to reproducibly bend within the BD and maintain its shape, allowing secure grasping of the stent’s anal side.

**Fig. 4:**
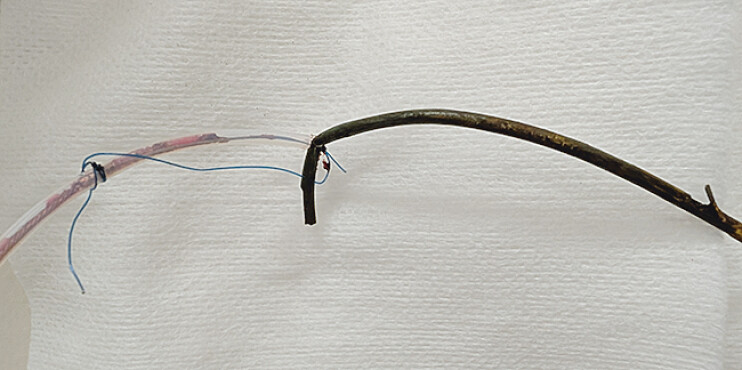
The migrated stent was securely grasped and successfully removed using the customized goose-neck snare.

Successful retrieval of a fully migrated and adherent biliary stent using a customized goose-neck snare with fixed-angle bending. Strong traction and precise control were achieved without specialized devices.Video 1

Manually bent snares have been reported; however, the curved shape often reverts upon sheath retraction 5 . Our method offers consistent bending and precise axial control. Snare size can be selected based on duct diameter, and no special equipment is required, making this a simple, reproducible approach.

Endoscopy_UCTN_Code_TTT_1AR_2AH

## Notes

Endoscopy E-Videos https://eref.thieme.de/e-videos
*E-Videos* is an open access online section of the journal *Endoscopy* , reporting on interesting cases and new techniques in gastroenterological endoscopy. All papers include a high-quality video and are published with a Creative Commons CC-BY license. Endoscopy E-Videos qualify for HINARI discounts and waivers and eligibility is automatically checked during the submission process. We grant 100% waivers to articles whose corresponding authors are based in Group A countries and 50% waivers to those who are based in Group B countries as classified by Research4Life (see: https://www.research4life.org/access/eligibility/ ). This section has its own submission website at https://mc.manuscriptcentral.com/e-videos .
